# ﻿Two new genera and one new species of the tribe Adeshini (Hymenoptera, Braconidae, Braconinae) from India and South Africa

**DOI:** 10.3897/zookeys.1166.105589

**Published:** 2023-06-12

**Authors:** Donald L. J. Quicke, Avunjikkattu Parambil Ranjith, Dharma Rajan Priyadarsanan, Mannankadiyan Nasser, Paul D. N. Hebert, Buntika A. Butcher

**Affiliations:** 1 Integrative Ecology Laboratory, Department of Biology, Faculty of Science, Chulalongkorn University, Bangkok 10330, Thailand Chulalongkorn University Bangkok Thailand; 2 Ashoka Trust for Research in Ecology and the Environment (ATREE), Royal Enclave, Srirampura, Jakkur Post, Bangalore 560064, India Ashoka Trust for Research in Ecology and the Environment (ATREE) Bangalore India; 3 Insect Ecology and Ethology Laboratory, Department of Zoology, University of Calicut, Kerala, Pin: 673635, India University of Calicut Calicut India; 4 Department of Integrative Biology, University of Guelph, Biodiversity Institute of Ontario, Guelph, Canada University of Guelph Guelph Canada

**Keywords:** 28S, Braconidae, COI, integrative taxonomy, phylogeny, new genera

## Abstract

Two new genera and one new species of the Braconinae tribe Adeshini are described and illustrated: *Crenuladesha* Ranjith & Quicke, **gen. nov.**, type species *Adeshanarendrani* Ranjith, 2017, **comb. nov.** from India, and *Protadesha* Quicke & Butcher, **gen. nov.**, type species *Protadeshaintermedia* Quicke & Butcher, **sp. nov.** from South Africa. The former lacks the mid-longitudinal propodeal carina characteristic of the tribe, and the latter displays less derived fore wing venation with two distinct abscissae of vein 2CU. A molecular phylogenetic analysis is included to confirm their correct placement. Since neither of the two new genera displays all of the characters given in the original diagnosis of the Adeshini a revised diagnosis is provided, as well as an illustrated key to the genera.

## ﻿Introduction

The subfamily Braconinae (Hymenoptera: Braconidae) is a diverse, species rich and morphologically distinct group of wasps with a cosmopolitan distribution (Shaw and Huddleston 1991). Until now, the subfamily included more than 2500 species belonging to 188 genera (Ranjith et al. 2016a, b). Most of the generic diversity is restricted to tropical regions (Quicke 2015) especially those of the Old World. The biology of braconines ranges from being strictly parasitoid, by far the commonest biology, though a few cases of entomophytophagy (Ranjith et al. 2016b), obligate phytophagy (Flores et al. 2005; Perioto et al. 2011) and obligate predation within plant galls are also known (Ranjith et al. 2022). Most braconines are idiobiont ectoparasitoids of immature hosts which principally are moderately concealed, such as gall inducers and leaf rollers or miners, or deeply concealed such as wood-borers (Shaw and Huddleston 1991; Quicke 1993; Ranjith et al. 2016b). Exceptionally, members of the Braconini subtribe Aspidobraconina are endoparasitoids within butterfly pupae (van Achterberg 1984; Quicke 1987) and this distinctive way of development was recently also reported in the apparently closely-related genus *Crinibracon* Quicke (Gupta et al. 2016).

The tribal classification of the Braconinae is confused, and has not been fully investigated. Historically, several tribes were erected based almost entirely on the West Palaearctic fauna (Quicke 1987). van Achterberg (1989) effectively synonymized several previously recognised tribes (see Quicke 1988b, 1990; Quicke et al. 1996), into the Aphrastobraconini (previously restricted to a small group of genera with aberrant wing venation, by defining them based on a ventrally elongate scapus. Formal changes in the systematic status of most of the remaining tribes have been superseded by molecular research (Belshaw et al. 2001) which supports two major clades, the Aphrastobraconini and the Braconini, and with the two genera of Coeloidini nested between them, though not as a monophyletic group. A detailed molecular study of relationships in preparation confirms that the Adeshini also constitute a valid separate tribe.

The tribe Adeshini was erected by van Achterberg (1983) within the Braconinae to accommodate two genera, *Adesha* Cameron and *Adeshoides* van Achterberg, which display a unique apomorphic condition of their fore wing venation with respect to the remainder of the subfamily, both having vein 2CU not differentiated into two abscissae and 2cu-a arising interstitially with m-cu (in the terminology of van Achterberg (1983) CUla arises at the same level as 2-CU1 and consequently having vein CUlb, if present, much longer than 3-CU1) (Quicke 1986).

Including those described in this paper, the Adeshini includes 16 described species classified within nine genera (van Achterberg 1983; Quicke 1986, 1988; Quicke and Ingram 1993; Quicke and Polaszek 2000; Wang et al. 2006; Liu et al. 2007; Ranjith et al. 2017; Oanh et al. 2023). The known species are entirely from the Old World, being distributed from Africa, through tropical Asia to Australia (Yu et al. 2016). The majority of the genera are known from only one or just a few specimens, and three previously described genera, *Adeshoides*, *Aneuradesha* and *Indadesha* are still monotypic (Quicke 1986; Quicke and Polaszek 2000). Further, most genera are currently known from more limited geographic regions, but *Africadesha* Quicke has a disjunct distribution comprising the Afrotropical and Australian regions (Quicke 1986; Quicke and Ingram 1993) and *Furcadesha* Quicke is recorded from tropical Asia to the Australia (Quicke 1986; Quicke and Ingram 1993; Liu et al. 2007).

Biology is known for four species, but based on these, they appear to be specialized parasitoids of hispine chrysomelid beetles. *Adeshaalbolineata* Cameron has been reared from the pest coconut leaf-miner, *Promecothecacumingi* Baly (Quicke and Polaszek 2000), *Aneuradeshaharleyi* Quicke from *Asmanguliacuspidata* Maulik, a minor pest of sugar-cane (Quicke and Polaszek 2000), and one species of *Furcadesha* has been reared from *Dactylispa* sp. (Jalala, unpublished), both on monocotyledonous host plants.

Here we describe two new, morphologically aberrant, genera of Adeshini to make their names available. We use DNA sequences from one mitochondrial (cytochrome oxidase I, COI) and one nuclear gene (28S) to confirm the placement of both new genera within the Adeshini. A revised tribe diagnosis and an illustrated key to all genera are presented. Further, we provide photographic portfolios of all of the adeshine genera.

## ﻿Materials and methods

### ﻿Sampling and morphology

The specimens of *Crenuladeshanarendrani* were collected by sweep netting and Malaise trapping from different localities of Kerala, India, and *Protadeshaintermedia* were collected by Malaise trapping from Gauteng, South Africa. Specimens from India were further dried with hexamethydisilazane and later card mounted. Measurements were taken from the holotypes and paratypes. Indian specimens were imaged using Keyence VHX-6000 digital microscope. *Protadeshaintermedia* gen. et sp. nov. was imaged using an Olympus SXZ16 microscope with automated multiple image capture at pre-set focal levels using an Olympus DP72 camera, and stacked using the Cell^D image processing system.

Morphological terminology used in the description follows van Achterberg (1979, 1988) except for wing venation which follows Quicke (2015). Body sculpture is described using the terminology of Harris (1979).

Collections housing specimens are abbreviated as follows:

**AIMB** ATREE Insect Museum, Bengaluru, India;

**CUMZ**Chulalongkorn University Museum of Zoology, Bangkok, Thailand;

**CNCO** Canadian National Collection of Insects and Arachnids, Ottawa, Canada;

**DZUC**Department of Zoology, University of Calicut, Kerala, India;

**FAFU** Beneficial Insects Laboratory, College of Plant Protection, Fujian Agriculture & Forestry University, China;

**IEBR**Braconidae Collection of the Department of Insect Ecology at the Institute of Ecology & Biological Resources, Vietnam Academy of Science and Technology, Ha Noi, Vietnam;

**KFRI**Kerala Forest Research Institute;

**MZLU**Museum of Zoology, Lund University, Lund, Sweden;

**NHMUK**Natural History Museum, London, U.K.;

**QDPI**Queensland Department of Primary Industries, Brisbane, Australia;

**QMBA**Queensland Museum, Brisbane, Queensland, Australia;

**SAMC**South African Museum, Cape Town, South Africa;

**ZJUH** Parasitic Hymenoptera Collection, Institute of Insect Sciences of the Zhejiang University, China.

### ﻿DNA extraction, amplification, and sequencing

DNA sequences were generated for the barcoding region of cytochrome oxidase c subunit 1 (COI), and the D2-D3 expansion region of 28S rDNA (28S) using standard protocols (Hajibabaei et al. 2005; Ivanova et al. 2006; de Waard et al. 2008). COI sequences were amplified using the following primers LepF1 (5’-GGT CAA CAA ATC ATA AAG ATA TTG G-3’) and LepR1 (5’-TAA ACT TCA GGG TGA CCA AAA AAT CA-3’) (Hebert et al. 2003, 2004; Park et al. 2010). Most 28S sequences were amplified using 28S-Fwd (5’-GCG AAC AAG TAC CGT GAG GG-3’) and 28S-Rev (5’-TAG TTC ACC ATC TTT CGG GTC CC-3’) (Belshaw and Quicke 1997); however, a few obtained prior to this study were amplified using D2B (5’-GTC GGG TTG CTT GAG AGT GC-3’) a variant on the primer D2-3549 (Campbell et al. 1993; Gauthier et al. 2000) and D3Ar (5’- TCC GTG TTT CAA GAC GGG TC-3’). Sequences are deposited on GenBank with accession numbers given in Table 1, and the aligned single gene datasets are provided in Suppl. material 1.

**Table 1.** Molecular voucher provenances, process IDs, barcode BINs, and GenBank accessions numbers for sequences.

Taxon	Provenance	BOLD process ID	BOLD BIN	GenBank accession number	
				CO1	28S
*Adesha* sp. 1	Thailand	ASQSP191-08	AAG7566	JF963429	ON128938
*Adesha* sp. 1	Indonesia	GMIAF056-17	AAG7566	ON325174	–
*Africadesha* sp.	Tanzania	ASQBR551-09	AAH8552	JF962472	ON256793
*Crenuladeshanarendrani*	India	DQHYM080-17	ADJ1989	MH260703	MH235025
*Furcadeshahuddlestoni*	India	DQHYM078-17	ADI8782	MH260692	MH235014
*Indadesha* sp.	India	DQHYM061-17	ADJ1754	MH26064	MH234965
*Protadeshaintermedia*	SouthAfrica	DQHYM029-17	ACK6473	MH260640	MH235000
*Spinadesha* sp.	Malaysia	GBAH1648-06	AAJ3611	AY935440	AY935440
*Acgoriumfelipechavarriai*	CostaRica	ASHYB1526-09	AAW2207	ON325243	OQ848754
*Bracongarugaphagae*	India	GBAHB1502-18	ADM1796	KT343804	KT343805
*Braconrosamondae*	Mexico	CNIN4339	–	ON324496	ON332043
*Braconella* sp.	Republic of Congo	BBTH1607-18	ADM1191	ON325113	OQ848752
*Carinibracon* sp.	India	BBTH2694-21	AEO3133	ON325239	ON129003
*Crinibraconchromusae*	India	DQHYM079-17	ADI5451	MH260687	MH235009
*Dolabraulax* sp.	India	DQHYM065-17	ADJ3321	ON256717	ON128994
*Eutropobraconindicus*	Thailand	BBTH809-17	ADH5940	MH260656	MH234975
*Habrobraconbrevicornis*	Thailand/Senegal	–	AAN5769	MF673598	MH766565
*Karposibraconpapuensis*	Papua New Guinea	BBTH1511-18	ADM3169	ON325001	ON128939
*Lyricibracon* sp.	Madagascar	GMMDH1498-15	ACS9968	MH260691	MH235013
*Myosoma* sp.	Peru	BBTH4919-22	AFA0865	OQ843071	OQ872371
*Physaraia* sp.	Thailand	BBTH2772-21	AEO3811	ON324889	ON128906
*Plesiobracon* group, gen. nov.	India	BBTH2715-21	AEO1467	ON325129	ON128972
*Sculptolobus* sp.	India	DQHYM057-17	ADJ2191	MH260655	MH234974
*Scutibraconhispae*	India	DQHYM068-17	ACY6119	ON324867	AJ296039
*Syntomernus* sp.	Bangladesh	GMBCJ1134-15	ADB6184	ON325202	ON128992
*Trigastrothecadoiphukhaensis*	Thailand	BBTH1811-19	AEC0115	ON325092	OQ848751
*Atanycolusulmicola*	USA	RRMFI4186-15	AAM4213	MG445079	ON128962
*Callibraconlimbatus*	Australia	GBAHB1501-18	–	EU106969	AJ231532
*Digonogastra* sp.	Honduras	GMHJJ537-15	ACS8451	MH260672	MH234994
*Euurobraconyokahamae*	Japan	BBTH2839-21	AEN9174	OL825724	OM950964
*Glyptomorpha* sp.	South Africa	KMPFT024-19	ADU9076	ON324905	OQ872372
*Iphiaulaximpostor*	Hungary	BBTH2613-21	ADA8959	ON325164	ON128983
*Pseudovipio* sp.	Tajikistan	GMIHG196-19	AEA3554	ON324906	OQ848750
*Rhammura* sp.	Uganda	ASQBR582-09	AAH6442	JF963807	MH308181
*Soter* sp.	Uganda	BBTH1544-18	AAG2535	ON324972	ON128929
*Stenobraconnicevillei*	Thailand	BBTH2756-21	AEN9387	ON325301	ON129028
*Coeloidessordidator*	Norway	COLHH1803-18	ADO6124	ON325175	OQ848753
*Colastesbraconius*	UK	ASQAS102-11	AAG1238	JF963124	HQ416429

A phylogenetic rapid bootstrap tree was generated using the maximum likelihood program RAxML (Stamatakis 2014).

### ﻿Phylogenetic analysis

A molecular data matrix comprising 37 Braconinae taxa plus one member of the Exothecinae was assembled from published and newly-generated sequences. Our taxon sampling was based on covering a reasonable amount of the taxonomic diversity of the Braconini and Aphrastobraconini. All genera were represented by both 28S D2 or D2+D3 sequences except for one *Adesha* specimen, and we included data from all available sequenced Adeshini specimens which collectively represented seven of the nine known genera.

The length-variable 28S sequences were aligned according to the secondary structure model of Gillespie et al. (2005) and our interpretation is provided in Suppl. material 1. The confidently alignable 28S bases were treated as either pairing or not-pairing (Butcher et al. 2014; Quicke et al. 2016), and the three codon positions of CO1 were also treated as separate partitions for analysis. Sequences were analyzed on RAxML (Stamatakis, 2014), using a GTR+G rate model with five data partitions (three cytochrome oxidase codons plus pairing and non-pairing 28S bases) and conducting a combined ML search and rapid bootstrap using the ‘-f a’ option and 100 runs.

## ﻿Results

### ﻿Phylogenetic results

**Figure 1.**
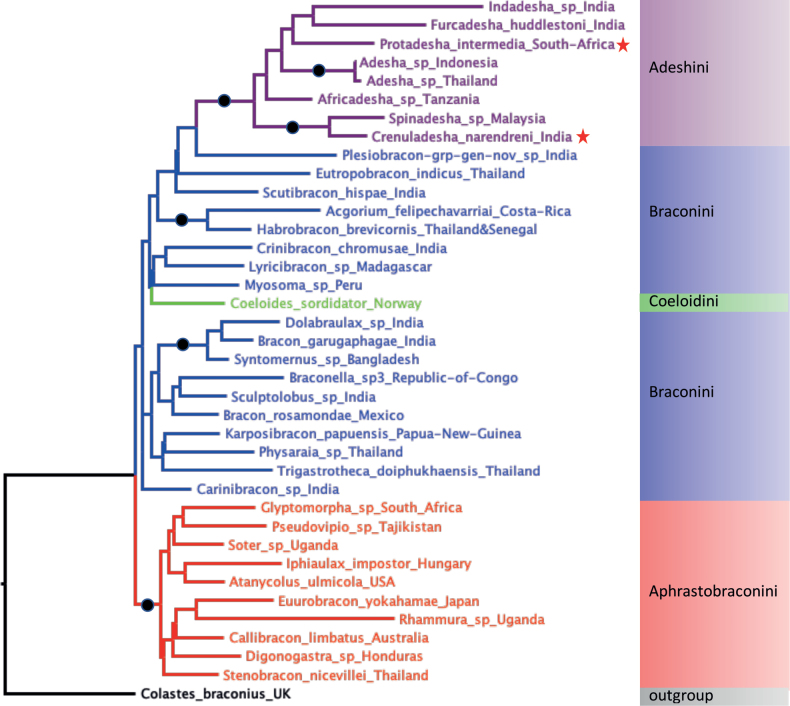
Maximum likelihood tree recovered from analyses of combined barcode and 28S D2-D3 rDNA sequence data showing major groupings and bootstrap support values. The two new taxa described are indicated by red star symbols. Nodes with 100% bootstrap support are indicated by black circles.

The Adeshini were recovered monophyletic with 100% bootstrap support as well as in individual analyses of the single gene data (not shown), but nested among the representative Braconini (as was the single included representative of the Coeloidini (Fig. 1). These three groups together formed a clade in the ML tree but with only 15% bootstrap support. The 10 included genera of Aphrastobraconini were recovered as a monophyletic clade with 100% bootstrap support (Fig. 1).

Both of the new genera described below were recovered in the Adeshini. *Crenuladesha* gen. nov. was recovered as sister group to *Spinadesha* with 100% bootstrap support. In neither of the single analaysis or combined trees was *Protadesha* gen. nov. recovered basally within the tribe (Fig. 1).

### ﻿Taxonomy

#### ﻿ Adeshini

Taxon classificationAnimaliaHymenopteraBraconidae

van Achterberg

BFD9B360-AE62-59A4-9484-06E792176D83

Adeshini van Achterberg, 1983: 175; Quicke 1987: 99; Quicke 1988a: 265.

##### Diagnosis.

Distinguished from other Braconinae by having the first abscissa of fore wing vein 2CU (the normally transverse vein 2CUa) short or completely absent, with 2CUb often arising at the same level of 1CUb OR if below the level of 1CUb, then vein 2cu-a present AND veins 2CU distinct AND the former not longer than vein 2cu-a. Additionally, scapus shorter ventrally than dorsally in lateral view; hind wing vein 1r-m very short, approximately as long as R, and oblique, the basal cell consequently being very narrow; hind wing posteriorly emarginate and setosity of posterior margin of hind wing very long compared with that of the wing membrane; first metasomal tergite movably connected to the second tergite; ovipositor shorter than the metasoma, at least with a distinct, pre-apical, dorsal angulation and ventral serrations.

##### Distribution.

Afrotropical, Australian, and Oriental regions.

##### Host.

Hispine chrysomelid beetles.

### ﻿Key to the genera of the tribe Adeshini van Achterberg

**Table:** 

1	Propodeum with mid-longitudinal groove which has large transverse crenulations posteriorly (Fig. 2F)	***Crenuladesha* Ranjith & Quicke, gen. nov.**
–	Propodeum without mid-longitudinal groove, with mid-longitudinal carina (usually complete but at least present on posterior half) (Figs 4F, 8B, C)	**2**
2	Fore wing vein 2CU differentiated into distinct transverse abscissa 2CUa which is approximately half as long as 2cu-a, followed by the longitudinal abscissa of 2CUb (Figs 4A, 5D, E); metasomal tergites 3–5 without transverse subposterior groove; first metasomal tergite without dorsal or mid-longitudinal carinae (Fig. 5A, C)	***Protadesha* Quicke & Butcher, gen. nov.**
–	Fore wing vein 2CU arising at the same level as 1CU, not divided into two distinct abscissae (Figs 6C, 8A, D, 9E) or if short basal abscissa present then it is longitudinal and in line with 2^nd^ part; metasomal tergites 3–5 with transverse subposterior grooves (Figs 7D, 8H, 9G); first metasomal tergite with dorsal or mid-longitudinal carina	**3**
3	Fore wing vein 2cu-a absent (Fig. 6C)	***Aneuradesha* Quicke**
–	Fore wing vein 2cu-a present (Figs 8A, 9E)	**4**
4	Posterior margin of fifth metasomal tergite multidentate (Fig. 7E), or with median pair of broad, flat projections (Fig. 7B, C)	**5**
–	Posterior margin of fifth metasomal tergite simple (Figs 8F, H, 9G) or only with posterolateral emarginations (Figs 8E, 9D)	**6**
5	Posterior margin of fifth metasomal tergite with multiple dentate projections (Fig. 7E); mesoscutum largely glabrous setose only along notaular line (Fig. 7D); median area of metanotum with simple mid-longitudinal carina; notauli crenulate only anteriorly	***Spinadesha* Quicke**
–	Posterior margin of fifth metasomal tergite with pair of fork-like projections medially (Fig. 7C); mesoscutum densely and evenly setose (Fig. 7A); median area of metanotum with distinct, lamelliform mid-longitudinal carina; notauli evenly crenulate	***Furcadesha* Quicke**
6	Laterope well developed, usually deep (Fig. 8H); mesosternum largely or entirely smooth (Fig. 8D, E); angle between fore wing veins C+SC+R and I-SR less than 50° (Fig. 8D)	**7**
–	Laterope absent; mesosternum coriaceous (Fig. 9A); angle between fore wing veins C+SC+R and I-SR more than 50°, often nearly 90° (Fig. 9A)	**8**
7	Mesoscutum with well-developed medioposterior pit and mid-longitudinal groove (Fig. 8B); 5^th^ metasomal tergite with well-developed posterolateral emarginations (see Figs 3E, 9D; mesoscutum largely densely setose (Fig. 8B)	***Adesha* Cameron**
–	Mesoscutum without medioposterior pit, without mid-longitudinal groove anteriorly, often rugulose medioposteriorly (Fig. 8C); 5^th^ metasomal tergite without or only with very weak posterolateral emarginations (Fig. 8E,F); mesoscutum largely glabrous or sparsely setose, the setosity being largely restricted to the vicinity of the notauli and medio-posterior region (Fig. 8C)	***Africadesha* Quicke**
8	Mesosternum coriaceous (Fig. 9B); second metasomal tergite without triangular mid-basal area and mid-longitudinal carina (Fig. 9C); 5^th^ metasomal tergite with well-developed posterolateral emarginations (Fig. 9D)	***Indadesha* Quicke**
–	Mesosternum smooth (Fig. 9F); second metasomal tergite with (small) triangular mid-basal area and mid-longitudinal carina (Fig. 9F); 5^th^ metasomal tergite without posterolateral emarginations (Fig. 9G)	***Adeshoides* van Achterberg**

#### ﻿ Crenuladesha

Taxon classificationAnimaliaHymenopteraBraconidae

Ranjith & Quicke gen. nov.

FA0E3C2B-F61A-5252-9978-B3D1ECBE008C

https://zoobank.org/DB1B942B-0724-4127-8E6F-80C7BB698134

##### Type species.

*Crenuladeshanarendrani* (Ranjith), comb. nov. by monotypy.

##### Diagnosis.

Differs from all other genera of Adeshini in having a complete, partially crenulate, mid-longitudinal propodeal groove, other genera having at least a partial, and usually complete, mid-longitudinal carina. In addition, unlike other members of the tribe the basal lobe of the claws is distinctly pectinate, and the dorsal valve of the ovipositor has a distinct pre-apical angulation in lateral profile, rather than being smoothly rounded.

##### Description.

***Head*.** Scapus shorter ventrally than dorsally in lateral aspect. Terminal flagellomere acute. Head transverse. Antennal sockets strongly produced in front of the eyes. Head smooth, setose. Malar suture distinct and complete. Frons depressed behind antennal sockets with mid-longitudinal groove. ***Mesosoma*.** Mesosoma smooth and densely setose. Mesoscutum without mid-longitudinal groove medio-posteriorly. Notauli crenulated, not meeting posteriorly. Scutellar sulcus crenulated. Scutellum smooth, setose. Median area of metanotum with complete mid-longitudinal carina. Mesopleuron smooth and setose, glabrous medially. Pleural suture smooth. Metasternum smooth. Propodeum with crenulated mid-longitudinal groove, with large crenulations posteriorly. ***Wings*.** Fore wings broad without infuscation. Second submarginal cell 2.50× as long as wide. Fore wing vein 2CUb longitudinal. Hind wing vein R longitudinal. Posterior margin of hind wing weakly emarginate. Base of hind wing without glabrous area next to vein cu-a. ***Legs*.** Tarsal claws with acute, pectinate basal lobe. ***Metasoma*.** Metasoma with five tergites, rugose. First metasomal tergite with medially united dorsal carina which extend to posterior margin in the form of a mid-longitudinal carina. Second metasomal tergite with smooth, triangular mid-basal area and a short mid-longitudinal carina, pair of posteriorly converging antero-lateral grooves. Second metasomal suture crenulate. Fifth metasomal tergite distinctly emarginated postero-laterally. Ovipositor with distinct dorsal angulation and ventral serrations.

##### Etymology.

From the combinations of Latin “crenula” meaning notched and *Adesha* type genus of the tribe, in reference to the crenulate propodeal groove.

##### Remarks.

The distinct and complete malar suture and crenulated mid-longitudinal groove on the propodeum are putative autapomorphies of *Crenuladesha* Ranjith & Quicke, gen. nov. In addition to that the presence of postero-lateral semicircular emarginations of the fifth metasomal tergite displays some affinities with the *Adesha*, *Furcadesha* and *Indadesha*. In common with *Adesha*, *Aneuradesha*, *Furcadesha* and *Spinadesha* are the presence of a mid-longitudinal groove on the frons, smooth vertex, mesosternum and pleural suture and exhibit a lesser character sharing with *Africadesha*, *Aneuradesha* and *Protadesha* on the basis of a single character; smooth mesosternum.

#### ﻿ Crenuladesha narendrani

Taxon classificationAnimaliaHymenopteraBraconidae

(Ranjith) comb. nov.

D0A23D4F-66F8-57FC-B5DE-568D6C18FE06

Figs 2 , 3

Adesha narendrani Ranjith, 2017: 101–103.

##### Type material.

***Holotype***, ♀, India, Kerala, Kozhikode, Janakikadu, 23.xii.2014, coll. Ranjith, A.P. (DZUC). ***Paratypes***, 1♀ same data as holotype; 1♀ and 1♂, India, Kerala, Malappuram, Nilambur, 5.iii.2015, coll. Ranjith, A.P.; 1♀, India, Kerala, Thrissur, Peechi (KFRI), 6.v.2015, coll. Ranjith, A.P.; 2 ♂, same data except 8.v.2015.

**Figure 2.**
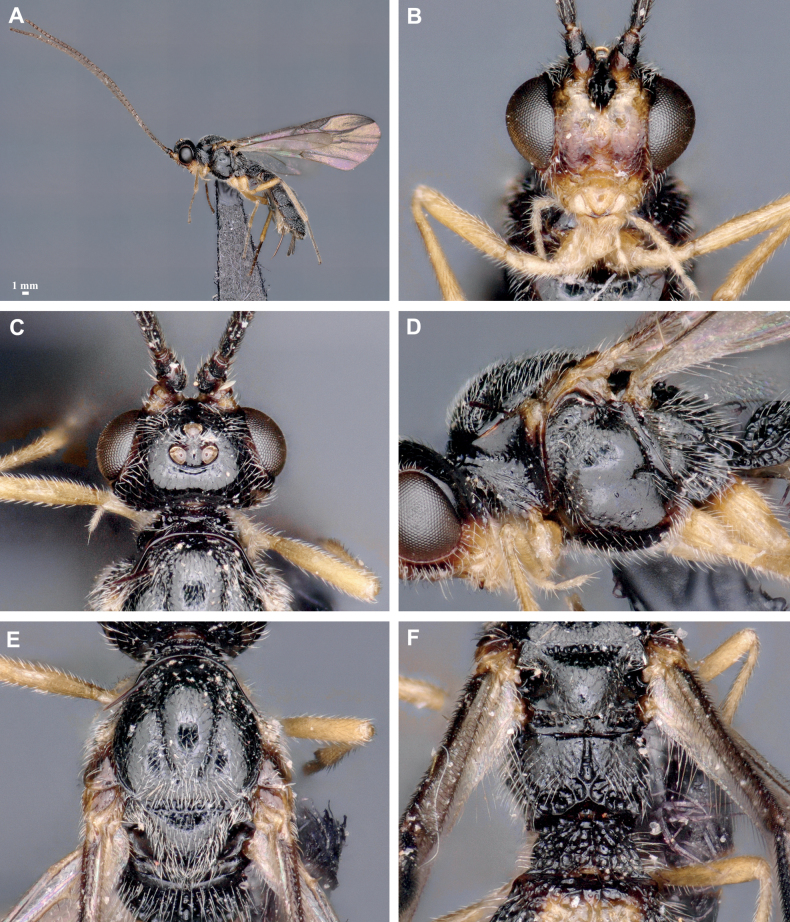
*Crenuladeshanarendrani*, ♀ **A** habitus, lateral view **B** head, anterior view **C** head, dorsal view **D** mesosoma, lateral view **E** mesoscutum and scutellum, dorsal view **F** propodeum and 1^st^ metasomal tergite, dorsal view.

##### Diagnosis.

The single species of the genus is distinguished from all other members of the tribe by its mid-longitudinally grooved propodeum.

**Figure 3.**
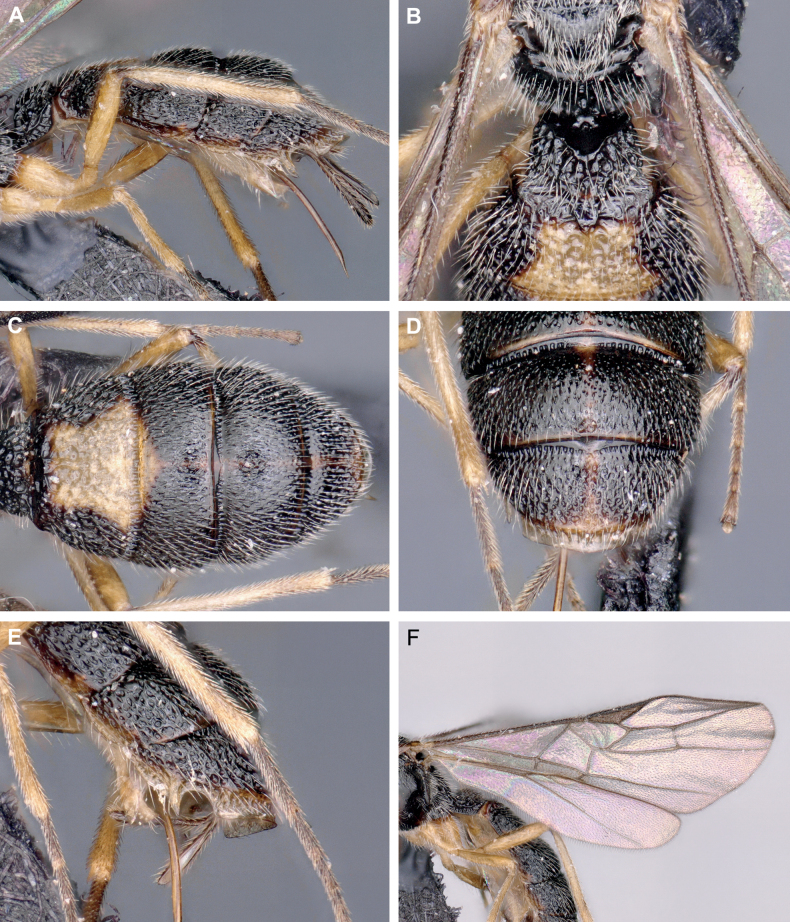
*Crenuladeshanarendrani*, ♀ **A** metasoma, lateral view **B** metasomal tergites 1 and 2, dorsal view **C** metasomal tergites 2–5, dorsal view **D** metasomal tergites 4 and 5, dorsal view **E** metasomal tergites 3–5, lateral view **F** wings.

##### Description

**(female).** Length of body 2.5 mm, of fore wing 2.2 mm, and of ovipositor (part exserted beyond apex of metasoma 0.3 mm. ***Head*.** Antennae with 33 flagellomeres. First flagellomere 1.10× and 1.20× as long as the second and third flagellomeres respectively. Third flagellomere 3.30× as long as wide. Terminal flagellomere acute. Height of clypeus: inter-tentorial distance: tentorio-ocular distance= 1.0: 2.2: 1.3. Face 0.50× as long as wide. POL: diameter of posterior ocellus: OOL= 1.0: 1.0: 2.5. Malar space 1.50× as long as basal width of mandible. ***Mesosoma*.** Mesosoma 1.40× as long as high. Pronotum protruding anteriorly and not distinctly emarginate in dorsal view, smooth anteriorly with fine striae posteriorly in lateral view. Notauli well developed and not running parallel, moderately converging posteriorly. Scutellar sulcus divided by seven carinae. ***Wings*.** Length of fore wing veins SR1: 3-SR: r = 8.2: 4.1: 1.0. Lengths of 2-SR: 3-SR: r-m = 1.6: 2.8: 1.0. Vein r as wide as 3-SR. Vein 2-SR slightly thinner than 3-SR. Hind wing vein SC+R1 2.00× as long as 1r-m. ***Legs*.** Length of fore femur: tibia: basitarsus= 1.7: 2.1: 1.0. Length of hind femur: tibia: basitarsus = 1.7: 2.8: 1.0. ***Metasoma*.** First metasomal tergite 1.30× wider posteriorly than long. Third metasomal tergite to fifth metasomal tergite sparsely punctate associated with setosity. Ovipositor sheath setose. Ovipositor 0.30× as long as hind tibia. ***Coloration*.** Reddish brown except mandibles, maxillary and labial palps, anellus, legs, second metasomal tergite medially and third metasomal tergite medio-basally yellow, face yellowish brown, tip of mandibles, pterostigma and venation brown.

**Male.** Similar to female.

##### Biology.

Unknown.

##### Distribution.

India (Kerala).

##### Variation.

The yellow coloration on the second metasomal tergite varies within paratypes. Eyes are silvery in one paratype.

#### ﻿ Protadesha

Taxon classificationAnimaliaHymenopteraBraconidae

Quicke & Butcher gen. nov.

208F14DE-E2A0-5F4F-B962-8BAC54E28CC3

https://zoobank.org/560D8706-AAAC-4BC2-8BEC-749A0F499A16

##### Type species.

*Protadeshaintermedia* Quicke & Butcher, sp. nov. by monotypy.

##### Diagnosis.

Differs from all other genera of Adeshini in having fore wing vein 2CU with a clearly-differentiated and sub-transverse basal abscissa (2CUa).

##### Description.

***Head*.** Scapus shorter ventrally than dorsally in lateral aspect. Head transverse. Head and mesosoma largely finely coriaceous or alutaceous. ***Mesosoma*.** Mesonotum with very weak narrow mid-longitudinal groove. Notauli narrow, complete, more or less uniting shortly before scutellar sulcus. Precoxal sulcus absent. Median area of metanotum with weak, mid-longitudinal carina. Propodeum with mid-longitudinal carina. ***Wings*.** Wings narrow. Fore wing second submarginal cell approximately 1.50× longer than wide. Fore wing vein 2CUa distinct. Hind wing vein 1r-m short, oblique. Hind wing vein R longitudinal. Posterior margin of hind wing weakly emarginate. Base of hind wing without glabrous area next to vein cu-a. ***Metasoma*.** Metasoma with five fully visible and sculptured tergites; tergites deep with steep sides. Metasomal tergites 3–5 without transverse subposterior grooves. First metasomal tergite not flattened laterally; with weak lateral depression along ventrolateral margin; with small, fenestrate dorsope. Second metasomal tergite without mid-basal or antero-lateral areas, with wide, weak lateral depression that runs along lateral margin as far as the base of third tergite. Second metasomal suture narrow, weakly curved. Fifth metasomal tergite very weakly emarginated posterolaterally. Ovipositor sharp, with a distinct pre-apical dorsal angulation (nodus).

##### Etymology.

From Greek *proto* meaning first and the genus name *Adesha* in reference to the potentially earlier form of modification of the fore wing venation in relation to that of other Adeshini.

##### Remarks.

The position of fore wing vein 2CUb half-way between the level of 2CU and the anal vein (hence with two angled abscissa of 2CU present) is intermediate between the usual condition in Braconinae and the derived state of the Adeshini, suggesting that the genus might be displaying a transitional character state.

#### ﻿ Protadesha intermedia

Taxon classificationAnimaliaHymenopteraBraconidae

Quicke & Butcher sp. nov.

0A3823CF-2787-57FE-9A4C-5DEFF767AA2C

https://zoobank.org/25308EB9-2BFC-4901-93EB-7A9FB96D4008

Figs 4 , 5

##### Type material.

***Holotype***, ♀. **South Africa**, Gauteng, Magaliesburg, Golden valley, private residence, 26.026°S, 27.545°E, 1526 m, coll. Herman Staude (CNCO). ***Paratype***, 1♀ same data as holotype (CNCO).

##### Description.

**Figure 4.**
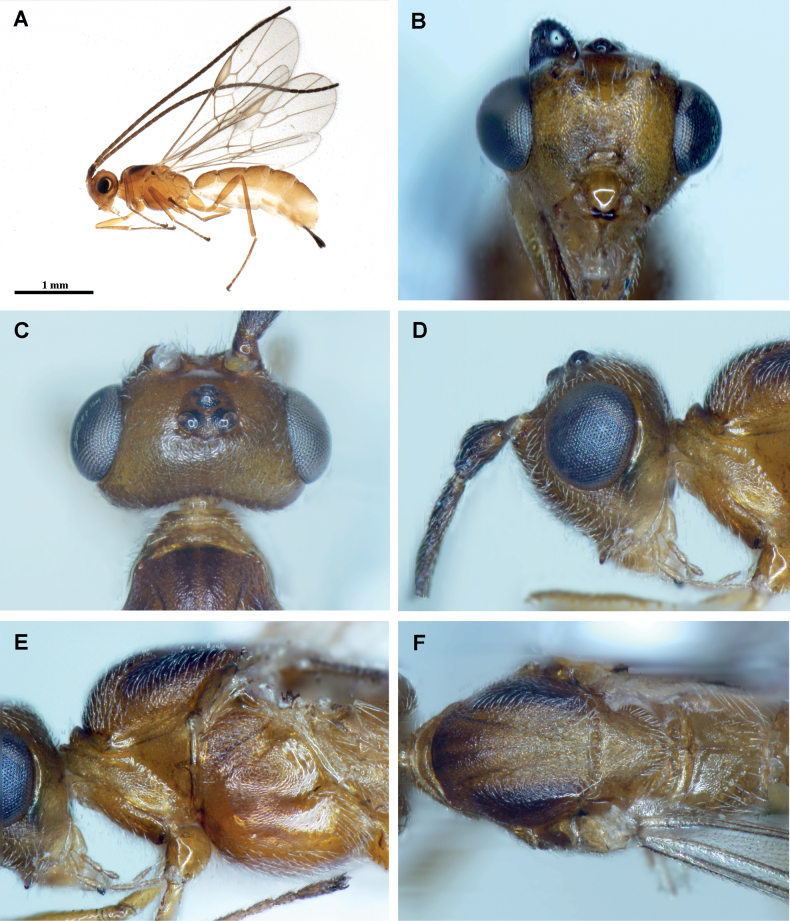
*Protadeshaintermedia* Quicke & Butcher, sp. nov., ♀, holotype **A** habitus, lateral view **B** head, anterior view **C** head, dorsal view **D** head, lateral view **E** mesosoma, lateral view **F** mesosoma, dorsal view.

Length of body 3.2 mm, of fore wing 2.7 mm and of ovipositor (part exserted beyond apex of metasoma 0.3 mm. ***Head*.** Antennae incomplete. Flagellomeres 1–3 approximately equal in length, 2.70× longer than wide. Width of head: width of face: height of eye = 3.3: 1.8: 1.0. Face very finely alutaceous with some weak oblique striation ventro-laterally. Inter-tentorial distance 1.4× tentorio-ocular distance. Malar space long; malar suture absent. Top of head finely coriaceous. Frons not impressed. Shortest distance between posterior ocelli: transverse diameter of posterior ocellus: shortest distance between posterior ocellus and eye = 1.8: 1.0: 2.0. Length of head behind eye 0.22× length of eye in dorsal view. Mesosoma 1.65× longer than high. Mesonotum finely coriaceous; notauli finely coriaceous. Mesopleuron weakly alutaceous dorso-laterally but otherwise largely shiny and glabrous. Mesosternum shiny but densely short setose. Propodeum with some irregular sculpture posteriorly. ***Wings*.** Lengths of veins r-rs: 3RSa: 3RSb = 1.0: 2.7: 6.8. Lengths of veins 2RS: 3RSa: rs-m = 1.4: 1.6: 1.0. Fore wing vein 2CUa distinct, approximately 0.6× length of 2cu-a. Hind wing vein 1M 7.0× longer than 1r-m. ***Legs*.** Lengths of fore femur: tibia: tarsus= 1.1: 1.0: 1.68. Lengths of fore femur: tibia: tarsus = 1.0: 1.5: 1.5. ***Metasoma*.** Metasomal tergites 1–5 alutaceous-coriaceous with some weak superimposed rugose sculpture laterally. First metasomal tergite without carinae. Second metasomal tergite 1.5× wider posteriorly than long; 1.2× longer than third metasomal tergite. Ovipositor sheathes approximately as long as hind basitarsus. **Coloration.** Body and legs uniformly dark honey brown except: three small dark marks, one on each lateral lobe and a smaller less distinct one medio-anteriorly on the medial lobe of the mesoscutum; tarsi of all legs which are darker brown. Wings with hyaline membrane and brown venation; pterostigma yellowish medially darker around its borders. It should be noted that the mounted holotype was chemically extracted for its DNA and is, as a consequence, is now somewhat duller than the fresh specimen would have been. Fig. 4A shows the specimen prior to DNA extraction.

**Male.** Unknown.

##### Biology.

Unknown.

##### Distribution.

South Africa (Gauteng).

##### Etymology.

The species name *intermedia* is Latin meaning in between, referring to the position of fore wing vein 2CUb relative to 1CU.

##### Comments.

DNA barcode cytochrome oxidase 1 and 28S D2-D3 expansion region sequences were obtained for the holotype (process ID = DQHYM029-17 sample ID = BIOUG36019-C05) and a barcode for the paratype (BIOUG09276-A04).

**Figure 5.**
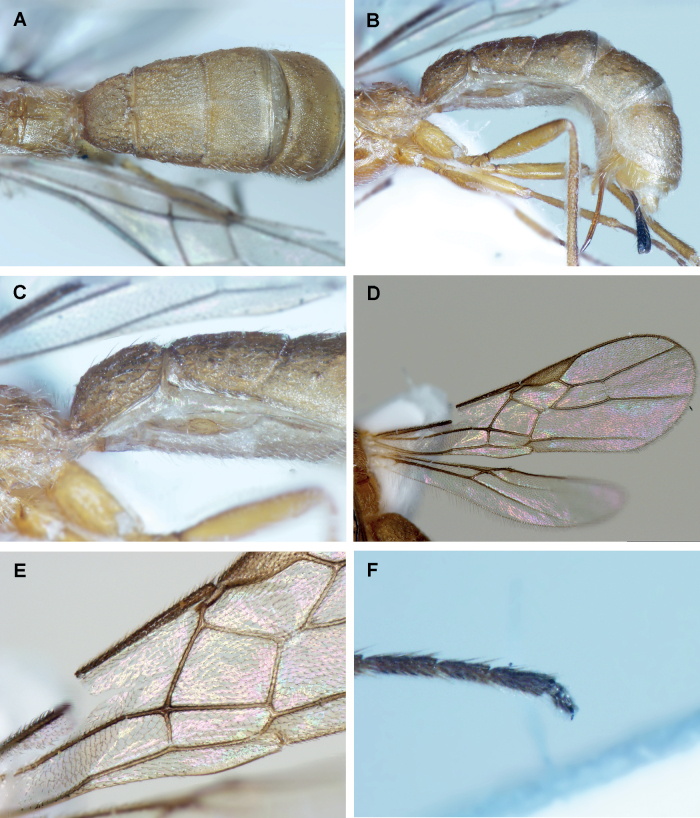
*Protadeshaintermedia* Quicke & Butcher, sp. nov., ♀, holotype **A** propodeum and metasoma, dorsal view **B** propodeum and metasoma, lateral view **C** metasomal tergites 1–3, lateral view **D** wings **E** middle part of fore wing detail **F** apex hind tarsus.

### ﻿Annotated list of species of Adeshini and additional material examined

***Adesha* Cameron, 1912**

Fig. 8A, B

*Adesha* Cameron, 1912: 1430. Type species *Adeshaalbolineata* Cameron, 1912.

**Distribution.** Thailand, Indonesia (Sumatra) (based on Global Malaise Trap project, process ID GMIAF056-17) and Pakistan. Note that the Thai and Sumatra specimens belong to the same DNA BIN, and are thus putatively conspecific.

***Adeshaalbolineata* Cameron, 1912**

**Type material. *Holotype*** ♀, Malaysia (Sarawak), Kuching, ?Feb. 1910 [“2.10”], coll. J. Hewitt (NHMUK).

**Distribution.** Malaysia (Sarawak), Pakistan (Nargis et al. 2020).

***Adeshaacuta* Quicke, 1986**

**Material examined. *Holotype*** ♀, Malaysia, Pahang, Kuala Lipis, 29.v.1926, coll. H.M. Pendlebury (NHMUK).

**Distribution.** Malaysia (Pahang), Pakistan (Nargis et al. 2020).

***Adeshoides* van Achterberg, 1983**

Fig. 9E–G

*Adeshoides* van Achterberg, 1983: 175. Type species *Adeshoidesasulcatus* van Achterberg, 1983.

**Distribution.** Senegal.

***Adeshoidesasulcatus* van Achterberg, 1983**

Fig. 9E–G

**Material examined** (via photographs). ***Holotype*** ♂, Senegal, 3 km S.S.W. Toubakouta, 10 km S. Ziguinchor, 4.iii.l977, at light, coll. Cederholm, Danielsson, Larsson, Mireström, Norling and Samuelson (MZLU).

**Distribution.** Senegal.

***Africadesha* Quicke, 1986**

Fig. 8C–H

*Africadesha* Quicke, 1986: 270. Type species *Africadeshausherwoodi* Quicke, 1986.

**Distribution.** Australia, Cameroun, Kenya, South Africa (based on unidentified species BOLD voucher BBTH4892-22), Tanzania (based on unidentified species BOLD voucher ASQBR551-09).

***Africadeshatobiasi* Quicke & Ingram, 1993**

Fig. 8D, E

**Material examined. *Holotype*** ♀, ***paratypes*** 3♀, Australia, Queensland, 15 km N.E. Kuranda, 1.v–14.vi.1985, coll. Storey & Halfpapp ("NHMUK", QDPI, QMBA).

**Distribution.** Australia (Queensland).

***Africadeshausherwoodi* Quicke, 1986**

Fig. 8C, F–H

**Material examined. *Holotype*** ♀, Cameroun, Nkoemvon, 19–30.xi.1979, coll. D. J. Jackson (NHMUK). **Additional material examined.** Kenya, Nyanza Province, Ungoye Down, 14–28.ix.2005, Malaise trap, coll. R. Copeland (CUMZ).

**Distribution.** Cameroon.

***Aneuradesha* Quicke, 2000**

Fig. 6

*Aneuradesha* Quicke, 2000: 104. Type species. *Aneuradeshaharleyi* Quicke, 2000 (in Quicke and Polaszek 2000).

**Distribution.** India, Pakistan.

***Aneuradeshaharleyi* Quicke, 2000**

Fig. 6

**Material examined. *Holotype*** ♂, ***paratype*** ♂, India, Uttar Pradesh, 8.vi.1998, coll. Atar Singh (NHMUK).

**Figure 6.**
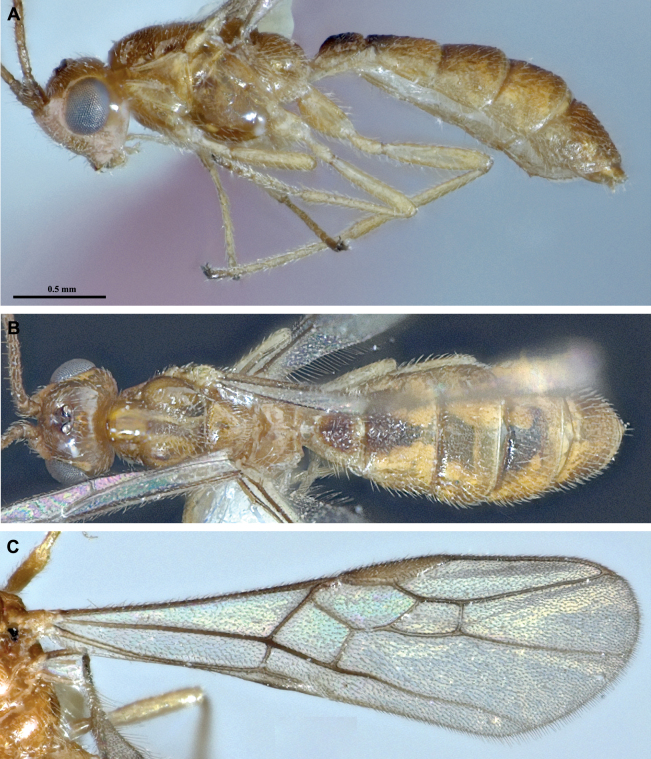
*Aneuradeshaharleyi* ♂ **A** habitus (excluding wings), lateral view **B** habitus (excluding wings), dorsal view **C** fore wing.

**Distribution.** India, Pakistan (Nargis et al. 2020).

***Furcadesha* Quicke, 1986**

Fig. 7A–C

*Furcadesha* Quicke, 1986: 266. Type species *Furcadeshahuddlestoni* Quicke, 1986.

**Figure 7.**
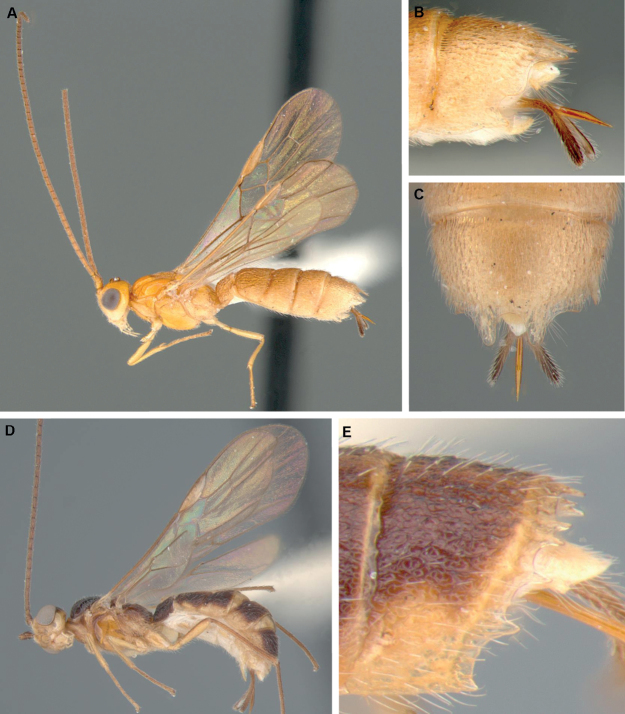
**A–C***Furcadeshawalteri*, ♀ **A** habitus, lateral view **B** metasomal tergite 5, lateral view **C** metasomal tergite 5, dorsal view **D, E***Spinadesha* sp., ♀ **D** habitus, lateral view **E** metasomal tergite 5, lateral view.

**Distribution.** China (Liu et al. 2007), India, Thailand, Australia

***Furcadeshabimaculatus* Yang & Liu, 2007**

**Type material. *Holotype*** ♀, China, (FAFU) [not examined].

**Distribution.** China (Guangxi).

***Furcadeshahuddlestoni* Quicke, 1986**

**Material examined. *Holotype*** ♀, India, Tamil Nadu, 4500 feet, 10.iv.1962, coll. D. J. Spencer (NHMUK). **Additional material examined.** 1 ♀, India, Kerala, Malappuram, Nilambur, 1.i.2016, coll. A.P. Ranjith. 1 ♀ Sri Lanka, Sigiriya, 7°57'N, 80°46'E, 18.iii.1999, coll. C. J. Burwell (AIMB).

**Distribution.** India, Sri Lanka (Ranjith personal observation).

***Furcadeshananningensis* Liu & Chen, 2007**

**Type material. *Holotype*** ♀, China, Nanning, Guangxi, 27.v.1988, coll. M.-Q. Zou (FAFU) [Not examined].

**Distribution.** China (Guangxi).

***Furcadeshawalteri* Quicke, 1986**

Fig. 7A–C

**Material examined. *Holotype*** ♀, Australia, Queensland, Mt. Nebo, 1.iv.1974, coll. I.D. Galloway (QMBA). **Additional material.** 2♀, Australia, Northern Territory, Darwin, off road, 13°13.02'S, 130°41.63'E, 21–27.viii.2004, K. Zhavrova (CUMZ).

**Figure 8.**
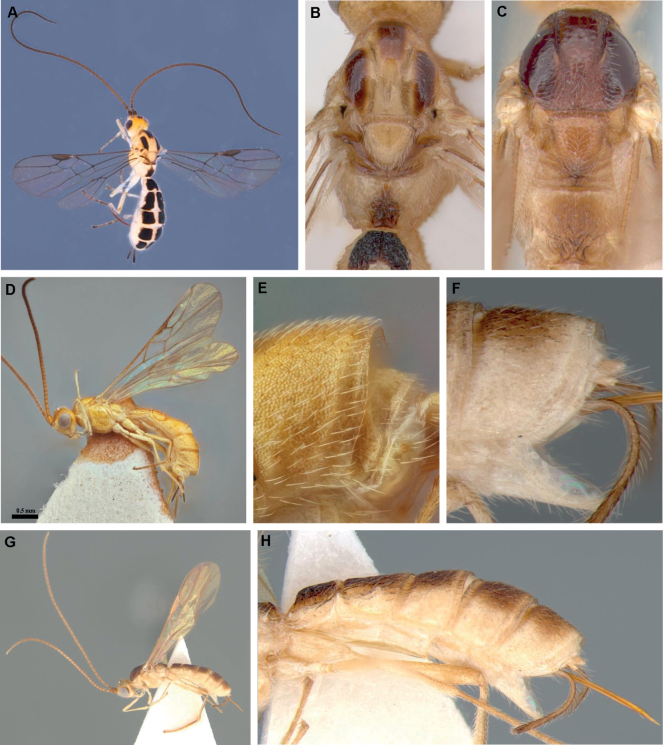
**A, B***Adesha* sp., ♀ **A** habitus, oblique dorsal view **B** mesosoma, dorsal view **C, F–H***Africadeshausherwoodi*, ♀ **C** mesosoma, dorsal view **F** metasomal tergite 5, lateral view **G** habitus, lateral view **H** metasoma, lateral view **D, E***Africadeshatobiasi*, ♀, holotype **D** habitus, lateral view **E** metasomal tergite 5, lateral view.

**Distribution.** Australia (Queensland).

***Indadesha* Quicke, 1986**

Figs 9A–D

*Indadesha* Quicke, 1986: 268. Type species *Indadeshaachterbergi* Quicke, 1986.

**Figure 9.**
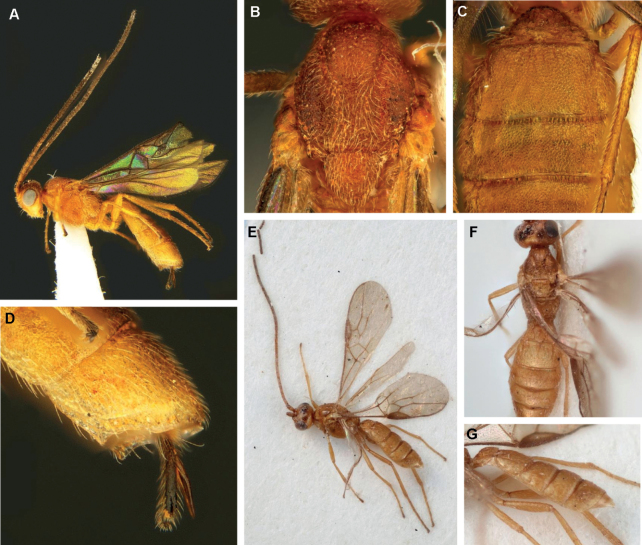
**A–D***Indadesha* sp., ♀ **A** habitus, lateral view **B** mesoscutum and scutellum, dorsal view **C** metasomal tergites 2 and 3, dorsal view **D** metasomal tergite 5, lateral view **E–G***Adeshoidesasulcatus*, ♂, holotype **E** habitus, lateral view **F** habitus, dorsal view **G** metasoma, lateral view.

**Distribution.** India.

***Indadeshaachterbergi* Quicke, 1986**

**Material examined. *Holotype*** ♀, India, Tamil Nadu, Shevaroy Hills, Yercaud, 4,500’, 1955, coll. P. S. Nathan (NHMUK).

**Distribution.** India.

***Spinadesha* Quicke**

FIg. 7D, E

*Spinadesha* Quicke,: 203. Type species *Spinadeshajacksoni* Quicke, 1988

**Distribution.** Thailand, Vietnam, China (Wang et al. 2006), and Malaysia (Sarawak).

***Spinadeshaalia* Long, 2023**

**Type material. *Holotype*** ♀, Vietnam, Quang Nam, Phuoc Son, forest, 15°26'N 107°53'E, 260 m, sweep net, 19.vii.2009, coll. K.D. Long (IEBR) [not examined].

**Distribution.** Vietnam.

***Spinadeshacrista* Long, 2023**

**Type material. *Holotype*** ♀, Vietnam, Quang Nam, Phuoc Son, forest, 15°26'N 107°53'E, 260 m, sweep net, 19.vii.2009, coll. K.D. Long (IEBR) [not examined].

**Distribution.** Vietnam.

***Spinadeshaintermedia* Long, 2023**

**Type material. *Holotype*** ♀, Vietnam, Ninh Binh, Cuc Phuong NP, Bong, forest, 20°20'50"N 105°35'47"E, 200 m, sweep net, 13.v.2005, coll. K.D. Long (IEBR) [not examined].

**Distribution.** Vietnam.

***Spinadeshajacksoni* Quicke, 1988**

**Material examined. *Holotype*** ♀, Thailand, Chang Mai, 14.viii-14.ix.1984, coll. D.J. Jackson ("NHMUK").

**Distribution.** Thailand.

***Spinadeshasinica* Wang et al., 2006**

**Type material. *Holotype*** ♀, China, Guang-dong, Chixingcheba Mountain, 24°40′29"N – 24°46′21"N, 114°09′04"E – 114°16′46"E), 25.V.2002, leg. Xu Zaifu, No. 20051439 (ZJUH). ***Paratypes***: 2♀, same data as holotype, Nos 20051285, 20051397 (ZJUH).

**Distribution.** China (Guangdong).

We have seen numerous other specimens of *Spinadesha* from Thailand and Borneo all of which are morphologically very similar. Oanh et al. (2023) provided a key to the five species described to date based largely on coloration and slight differences in the profiles of the denticulations on the posterior margin of the 5^th^ metasomal tergite.

## ﻿Discussion

### ﻿Morphological plasticity

Although with only weak bootstrap support, the combined analysis (Fig. 1) and each individual gene analysis (not shown) recovered both *Crenuladesha* gen. nov. and *Protadesha* gen nov. in derived positions within the Adeshini. This suggests that the presence of a sub-transverse fore wing vein 2CU1b in *Protadesha* is likely a character reversal. The arrangement of veins forming the distal part of the 1^st^ subdiscal cell with 1CU and 2CU at the same level as in other Adeshini occurs in several other groups of Braconidae, e.g., some Hormiinae, the rogadine tribe Facitorini, a few Doryctinae and Alysiinae. In all these cases it is restricted to small-bodied species/genera. The longitudinal propodeal groove of *Crenuladesha* seems likely to have resulted from increasing depth of the often crenulate border of the mid-longitudinal carina, finally with the central carina being lost. During such an evolutionary transition, the function of the structure (presumably propodeal strengthening) would be maintained.

Even though many of the genera are represented by one or two species, both *Furcadesha* and *Africadesha* have particularly wide distributions. Several species, based on morphology, also appear to have wide distributions, for example, *Adeshaalbolineata* (Borneo to Pakistan) and *A.acuta* (Peninsular Malaysia to Pakistan). Further, two specimens of *Adesha* sp., one from Thailand the other from the island of Sumatra (Indonesia) for which DNA sequence data are available, had barcodes belonging to the same BIN (AAG7566) and thus are most probably conspecific.

## Supplementary Material

XML Treatment for Adeshini

XML Treatment for Crenuladesha

XML Treatment for Crenuladesha narendrani

XML Treatment for Protadesha

XML Treatment for Protadesha intermedia

## ﻿Additional information

### Author ORCIDs

Donald L.J. Quicke https://orcid.org/0000-0003-4471-6775

Avunjikkattu Parambil Ranjith https://orcid.org/0000-0001-7061-9659

Dharma Rajan Priyadarsanan https://orcid.org/0000-0001-8137-3404

Mannankadiyan Nasser https://orcid.org/0000-0002-6460-1839

Paul D.N. Hebert https://orcid.org/0000-0002-3081-6700

Buntika A. Butcher https://orcid.org/0000-0002-0541-0709

### Data availability

All of the data that support the findings of this study are available in the main text or Supplementary Information.

## Supplementary materials

Supplementary material 1 Molecular data sets used for phylogenetic analysis Data type Molecular data sets used for phylogenetic analysis Explanation note Molecular data sets used for phylogenetic analysis, and for the length-variable 28S rDNA gene, also our interpretation of secondary structure; pairing bases indicated by ‘p’, non-pairing by ‘u’ and ambiguously alignable ones not included in the analyses, by ‘x’. https://binary.pensoft.net/file/862724This dataset is made available under the Open Database License (http://opendatacommons.org/licenses/odbl/1.0/). The Open Database License (ODbL) is a license agreement intended to allow users to freely share, modify, and use this Dataset while maintaining this same freedom for others, provided that the original source and author(s) are credited.Donald L.J. Quicke, Avunjikkattu Parambil Ranjith, Dharma Rajan Priyadarsanan, Mannankadiyan Nasser, Paul D.N. Hebert, Buntika A. Butcher
